# Childhood-Onset Schizophrenia: A Systematic Overview of Its Genetic Heterogeneity From Classical Studies to the Genomic Era

**DOI:** 10.3389/fgene.2019.01137

**Published:** 2019-12-18

**Authors:** Arnaud Fernandez, Malgorzata Marta Drozd, Susanne Thümmler, Emmanuelle Dor, Maria Capovilla, Florence Askenazy, Barbara Bardoni

**Affiliations:** ^1^University Department of Child and Adolescent Psychiatry, Children’s, Hospitals of NICE CHU-Lenval, Nice, France; ^2^CoBTek, Université Côte d’Azur, Nice, France; ^3^Université Côte d’Azur, CNRS UMR7275, Institut de Pharmacologie Moléculaire et Cellulaire, Valbonne, France; ^4^Université Côte d’Azur, INSERM, CNRS UMR7275, Institut de Pharmacologie Moléculaire et Cellulaire, Valbonne, France

**Keywords:** childhood-onset schizophrenia, autism spectrum disorder, genetics, copy number variations, single nucleotide polymorphisms, exome sequencing

## Abstract

Childhood-onset schizophrenia (COS), a very rare and severe chronic psychiatric condition, is defined by an onset of positive symptoms (delusions, hallucinations and disorganized speech or behavior) before the age of 13. COS is associated with other neurodevelopmental disorders such as autism spectrum disorder (ASD) and attention deficit and hyperactivity disorder. Copy number variations (CNVs) represent well documented neurodevelopmental disorder risk factors and, recently, *de novo* single nucleotide variations (SNVs) in genes involved in brain development have also been implicated in the complex genetic architecture of COS. Here, we aim to review the genetic changes (CNVs and SNVs) reported for COS, going from previous studies to the whole genome sequencing era. We carried out a systematic review search in PubMed using the keywords “childhood(early)-onset schizophrenia(psychosis)” and “genetic(s) or gene(s) or genomic(s)” without language and date limitations. The main inclusion criteria are COS (onset before 13 years old) and all changes/variations at the DNA level (CNVs or SNVs). Thirty-six studies out of 205 met the inclusion criteria. Cytogenetic abnormalities (n = 72, including 66 CNVs) were identified in 16 autosomes and 2 sex chromosomes (X, Y), some with a higher frequency and clinical significance than others (e.g., 2p16.3, 3q29, 15q13.3, 22q11.21 deletions; 2p25.3, 3p25.3 and 16p11.2 duplications). Thirty-one single nucleotide mutations in genes principally involved in brain development and/or function have been found in 12 autosomes and one sex chromosome (X). We also describe five SNVs in X-linked genes inherited from a healthy mother, arguing for the X-linked recessive inheritance hypothesis. Moreover, *ATP1A3* (19q13.2) is the only gene carrying more than one SNV in more than one patient, making it a strong candidate for COS. Mutations were distributed in various chromosomes illustrating the genetic heterogeneity of COS. More than 90% of CNVs involved in COS are also involved in ASD, supporting the idea that there may be genetic overlap between these disorders. Different mutations associated with COS are probably still unknown, and pathogenesis might also be explained by the association of different genetic variations (two or more CNVs or CNVs and SNVs) as well as association with early acquired brain lesions such as infection, hypoxia, or early childhood trauma.

## Introduction

Childhood-onset schizophrenia (COS) is a rare (< 1/40,000) and severe chronic psychiatric condition that shares with adult-onset schizophrenia (AOS) positive symptoms (delusions, hallucinations, and disorganized speech or behavior), but presents an early onset (before the age of 13) (Burd and Kerbeshian, 1987; Nicolson and Rapoport, 1999). It remains considered by many authors as an early and severe variant of AOS (Nicolson and Rapoport, 1999; Biswas et al., 2006). In COS, neurodevelopmental abnormalities (deficits in cognition, communication, or neuromotor impairments) and premorbid dysfunction are more frequent compared with AOS (Vourdas et al., 2003) and a clinical overlap exists with other neurodevelopmental disorders: 28% of patients with COS in the US cohort of the National Institute of Mental Health Child Psychiatry Branch met criteria for comorbid autism spectrum disorder (ASD) (Rapoport et al., 2009). In addition, more than 80% of children with schizophrenia or schizoaffective disorder present comorbid attention deficit and hyperactivity disorder (ADHD) (Ross et al., 2006). Few genetic studies of COS were reported, due to the very low prevalence (<1/40,000) (Burd and Kerbeshian, 1987) and to nosographic difficulties, which made it hard to obtain a consensual clinical definition of this disorder and to carry out etiological studies (Maier, 1999; Gochman et al., 2011). Diagnostic and Statistical Manual of Mental Disorders, Fifth Edition (DSM-5) classification provides recent clarification in this area with schizophrenia no longer excluding the diagnosis of ASD (Petty et al., 1984; American Psychiatric Association, 2013). Thus, clinical overlap between COS and ASD is now formally accepted. Surprisingly, DSM-5 still does not recognize the existence of COS, which therefore remains considered an adult clinical presentation (AOS) (American Psychiatric Association, 2013). Indeed, COS is a very rare complex disorder related to other neurodevelopmental disorders, and it represents a real challenge for clinical diagnosis with, to date, no objective test based on genetics (Petty et al., 1984). However, a high heritability rate of COS (> 80%) has been suggested in early adoption/twin studies (Kallmann and Roth, 1956) and has been confirmed by familial aggregation studies (Asarnow and Forsyth, 2013). To determine the etiology of COS, it is indispensable to start by reviewing the publications that have linked COS to DNA changes.

Macro-lesional cytogenetic abnormalities such as copy number variations (CNVs), including the 22q11.21 deletion, are more frequent in COS than in AOS [10.6% of patients with COS (DSM-III-R, onset <13 years) vs. 2–5%, in AOS, p < 0.0001]. These anomalies in the general population would concern only 0.86% of newborns (Nicolson et al., 1999).

Recently, Next Generation Sequencing (NGS) or “high throughput sequencing” allowed, with unprecedented scalability and speed, to determine the DNA sequence of a given individual. This tool opened up new perspectives to understand complex neurodevelopmental disorders, with particular attention to *de novo* single nucleotide variations (SNVs) occurring in genes involved in brain development (Veltman and Brunner, 2012). Only one study used whole exome sequencing (WES), a NGS method, in a cohort of patients with COS. This study identified 20 *de novo* variants in 17 COS probands (rate: 1.17) in genes previously linked to neuronal function or to psychiatric disorders (Ambalavanan et al., 2016). These arguments (phenotypic overlap with other neurodevelopmental disorders, high heritability, disease-related CNVs, and *de novo* SNV rates) strongly support the neurodevelopmental and genetic bases of COS (Rapoport et al., 2012). In this context, the main aim of this study is to describe the COS genomic variation (CNVs and SNVs) in the scientific literature to identify interesting genes or genetic pathways in both clinical practice and research.

## Methods

We carried out a systematic review of the MEDLINE database accessible *via* the search engine PubMed (www.ncbi.nlm.nih.gov/pubmed/) with the following key words: “childhood-onset schizophrenia” or “childhood-onset psychosis” or “early-onset schizophrenia” or “early-onset psychosis” and “genetics” or “genetic” or “gene” or “genes” or “genomic” or “genomics.” Our search terms were not limited by language or date of publication and were manually reviewed. According to inclusion criteria, we considered all genomic changes occurring in COS patients (age of onset before 13). We excluded all abnormalities at RNA or protein levels (regardless the age of onset). Genomic variations were classified based on cytogenetic position (**Table 1**) and candidate gene names (**Table 2**).

**Table 1:** Cytogenetic abnormalities (including copy number variations) in COS patients with their localization, length and type of inheritance.

CHR	CNV region and type (length)	Sex, age of onset	Neurodevelopmental disorders, Comorbidities	Inheritance	coordinates (hg18-*hg19*)	SFARI	Clinical significance	Bibliography
1	DUP 1q21.3 (248 kb)	Male, 12 y	−	inherited	chr1:151,514,380-151,762,871	+	UCS	Walsh et al., 2008
1 ; 7	t(1;7) (p22;q22)	Male, 9 y	ASD? - ADHD? - language, intellectual and motor impairments, dysmorphia, supraventricular tachycardia	inherited (father)	−	−	−	Gordon et al., 1994; Nicolson et al., 1999; Yan et al., 2000; Idol et al., 2008; Eckstrand et al., 2008; Addington and Rapoport, 2009*
2	DUP 2p14 (243 kb)	<13 y	−	inherited	chr2:65,637,097-65,879,935	+	UCS	Walsh et al., 2008
	DEL 2p16.3 (115 --> 112 kb) NRXN1	<13 y	−	not known	chr2:50,023,212-50,137,825 / chr2:50,025,162-50,136,989	+	P	Walsh et al., 2008; Addington and Rapoport, 2009*, Ahn et al., 2014
	DEL 2p16.3 (38 et 40kb) NRXN1	Male, 12 y	Motoric and verbal delay, IQ 82, macrocencephalia and increase height (+3SD)	inherited (father)	chr2:51,151,955-51,190,352 / chr2:51,440,969-51,481,281	+		Duong et al., 2015
	DUP 2p25.3 (216 --> 245 kb) MYT1L	<13 y	−	inherited (mother)	chr2:1,618,945-1,835,426 / chr2:1,591,064-1,836,375	+	P	Walsh et al., 2008; Addington and Rapoport, 2009*, Ahn et al., 2014; Lee et al., 2012
	DUP 2p25.3 (143 --> 107kb ) MYT1L	<13 y	−	not known	chr2:1,713,636-1,857,129 / chr2:1,720,133-1,827,317			
	DEL 2q31.2-31.3 (2,5 Mb)	Male, 11 y	−	de novo or germline mosaicism in one of the parents	chr2:179,643,864-182,145,339	-	UCS	Walsh et al., 2008; Addington and Rapoport, 2009*
3	DEL 3p12.2-p12.1 (2,2 Mb)	Female, 12 y	ASD, poor motor coordination, IQ 67	inherited (father)	−	-	UCS	Rudd et al., 2015
	DUP 3p21.31 (117 Kb)	<13 y	−	inherited	chr3:45,458,901-45,576,135	+	UCS	Walsh et al., 2008
	DUP 3p25.3 (120 --> 134 kb) SRGAP3	Male, 11 y	poor peer relationships, general anxiety disorder, panic disorder, agoraphobia, and depression	inherited (father)	chr3:9,100,744-9,220,529 / *chr3: 9,111,177–9,245,155*	+	P	Walsh et al., 2008; Addington and Rapoport, 2009*, Wilson et al., 2011
	DEL 3q29 (1,58 Mb)	Male, 5 y	ASD, severe abdnormal movements and tics	de novo	chr3:197,161,073-198,851,029	+	P	Sagar et al., 2013
5	DUP 5q12.3 (142 Kb)	Female, 10 y	−	inherited	chr5:64,795,287-64,937,409	+	UCS	Walsh et al., 2008
	Paternal segmental iUPD 5q32-qter (35 Mb)	Female, 9 y	MDD, inattention,learning disability, intellectual impairments	*de novo*	−	−	−	Eckstrand et al., 2008; Addington and Rapoport, 2009*, Seal et al., 2006
6	DEL 6p22.31 (144 kb)	Male, 9 y	OCD, expessive language disorder	inherited	chr6:119,596,633-119,740,850	+	UCS	Walsh et al., 2008
7	DUP 7p13 (120 Kb)	<13 y	−	not known	chr7:44,420,900-44,540,491	-	UCS	Walsh et al., 2008
	DUP 7q11.21-q11.22 (2,8 Mb)	<13 y	−	inherited	chr7:64,126,564-66,883,376	+	UCS	
8	DUP 8p22 (1,3 Mb)	Male, 11 y	−	inherited	chr8:13,400,795-14,679,483	+	UCS	Walsh et al., 2008
	DUP 8q11.23 (480 kb --> 493 kb)	<13 y	−	not known	chr8:53,563,161-54,043,063 / chr8:53,550,992-54,043,684	+	UCS	Walsh et al., 2008; Ahn et al., 2014
	DUP 8q24.3 (369 Kb) PTK2	12 y	−	not known	chr8:142,025,432-142,393,948	+	UCS	Walsh et al., 2008
9	DEL 9p24.2 (440 Kb)	11 y	−	−	chr9:3,104,250-3,544,339	+	UCS	
10	DUP 10p11.23 (176 Kb)	Male, 11 y	−	inherited	chr10:28,990,284-29,166,175	+	UCS	Walsh et al., 2008
	DUP 10p13 (145 Kb)	<13 y	−	inherited	chr10:15,688,654-15,833,865	+	UCS	
	DEL 10q22.3 (173 kb)	Male, 12 y	−	*de novo*	chr10:81,415,378-81,588,866	+	UCS	Ahn et al., 2014
	DEL 15q11.2 (1386 kb)	<13 y	−	not known	chr15:18,818,086-20,203,694	+	UCS	Ahn et al., 2014
15	DEL 15q11.2 (575kb)	<13 y	−	inherited (mother)	chr15:20,203,​694-20,778,963			
	DEL 15q13.3 (382kb)	<13 y	−	*de novo*	chr15:30,238,780-30,620,951	+	P	
	DEL 15q13.3 (475 kb)	<13 y	−	inherited (mother)	chr15:30,238,780-30,713,368			
	DUP 15q13.3 (503.5 Kb) CHRNA7	Female, 10 y	−	inherited (father)	chr15:32,012,361-32,515,849	+		Zhou et al., 2016
	DUP 15q13.3 (600,2 Kb) CHRNA7	Male, 12 y	ADHD	chr15:32,019,919–32,620,127				
	DUP 15q26.2-q26.3 (687 Kb)	<13 y	−	not known	chr15:96,246,764-96,933,404	+	UCS	Walsh et al., 2008
16	DUP 16p11.2 ( 433 --> 604 Kb)	8 y	PDD-NOS, poor social and motor development	inherited (father)	chr16:29,652,656-30,085,308 / chr16:29,502,984-30,107,306	+	P	Walsh et al., 2008; Addington and Rapoport, 2009*, Ahn et al., 2014; Rapoport et al., 2009
	DUP 16p11.2 ( 578 --> 445 kb)	10 y	Poor social and motor development	chr16:29,657,405-30,235,818 / chr16:29,782,436-30,227,808				
	DEL 16p12.1 (449kb)	−	−	father	chr16:21,498,074-21,946,841	+	UCS	Ahn et al., 2014
	DEL 16p13.11 (15 à 131 kb)	Male, 6 y	Motor dyscoordination, langage impairments	Father or de novo	*chr16:1,51,32,264–1,51,47,411 (min) to 1,50,48,733-1,51,79,946 (max)*	*+*	UCS	Brownstein et al., 2016
	DUP 16p13.11 (1,4 à 1,7 Mb)	Female, 4 y	ASD, Epilepsy, Chiari 1	father	*chr16:1,48,97,761–1,62,76,117 to 1,47,80,303–1,64,58,270*		UCS	
	DUP 16q22.2-ter (17 Mb)	Female, 11 y	Atypical Turner, motor, language and attention impairments	−	−	−	P	Eckstrand et al., 2008
	DUP 16q22.3-q24.3 (16,7 Mb)	Female, 12 y	ASD, poor motor coordination, IQ 67	*de novo*	−	−	P	Rudd et al., 2015
	DUP 16q23.3 (1,5 Mb)	Female, 9.5 y	−	inherited	chr16:80,737,839-82,208,451	+	UCS	Walsh et al., 2008
	DEL 16q24.1 (111 Kb)	<3 y	−	inherited	chr16:82,997,582-83,108,554	+	UCS	
17	DUP 17q21.31 (384 kb)	<13 y	−	father	chr17:41,321,621-41,706,070	+	UCS	Ahn et al., 2014
18	DUP 18p11.31-p11.23 (510 kb)	<13 y	−	inherited	chr18:7,067,237-7,576,777	+	UCS	Walsh et al., 2008
	DUP 18q22.1 (768 Kb)	Male, 10 y	Asperger's disorder	inherited	chr18:61,907,915-62,675,869	+	UCS	Walsh et al., 2008
19	DEL 19p12 (397 Kb)	<13 y	−	not known	chr19:23,413,380-23,810,606	+	UCS	Walsh et al., 2008
20	DEL 20p12.1 (113 Kb)	10 y	Poor social and motor development	inherited	chr20:14,921,777-15,034,862	+	UCS	Walsh et al., 2008
22	DEL 22q11.2 (3Mb) PRODH DGCR6	Male, 9 y	Language, motor and social impairments, generalized anxiety disorder, dysthymia and ADHD, craniofacial dysmorphia, hypospadias	de novo	−	+	P	Nicolson et al., 1999; Eckstrand et al., 2008; Addington and Rapoport, 2009*, Rapoport et al., 2009; Yan et al., 1998; Usiskin et al., 1999; Liu et al., 2002; Sporn et al., 2004
		Female, 12 y	Language, motor and social impairments, craniofacial dysmorphia, celiac disease and ureteric reflux	de novo	−			Nicolson et al., 1999; Eckstrand et al., 2008; Addington and Rapoport, 2009*, Rapoport et al., 2009; Usiskin et al., 1999; Liu et al., 2002; Sporn et al., 2004
		Female, 10 y	Language, motor and social impairments, craniofacial dysmorphia	de novo	−			
		<13 y	Craniofacial dysmorphia	−	−			Eckstrand et al., 2008; Addington and Rapoport, 2009*, Rapoport et al., 2009; Sporn et al., 2004
	DEL 22q11.21 (3 Mb)	<13 y	−	*de novo*	chr22:17,092,563-20,077,678	+		Ahn et al., 2014
	DEL 22q11.21 (2,6 Mb)	<13 y	−	*de novo*	chr22:17,224,632-19,842,333			
	DEL 22q11.21 (2,6 Mb)	<13 y	−	not known	chr22:17,257,787-19,855,248			
	DEL 22q11.21 (2,7 Mb)	<13 y	−	*de novo*	chr22:17,257,787-19,963,350			
	DEL 22q11.21 (2,9 Mb)	<13 y	−	*de novo*	chr22:17,269,794-20,128,199			
	DUP 22q13.32 (1,6 Mb)	8 y	PDD-NOS, poor social and motor development	*de novo*	chr22:47,903,228-49,557,485	+	UCS	Ahn et al., 2014
X	expansion CGG (1,5 Kb) FMR1	Female, 9 y	Dysmorphia, learning and social impairments, mild MR	mother	−	−	−	Vantalon et al., 2005
	47, XXX	Female, <13 y	−	−	−	−	−	Eckstrand et al., 2008; Addington and Rapoport, 2009*
	46,X,i(X)(q10)(22%)/45,X(78%)	Female, <13 y	Mosaic Turner	*de novo*	−	−	−	
	DEL Xq24-qter	Female, 11 y	Atypical Turner, motor, language and attention impairments	*de novo*	−	-	P	Nicolson et al., 1999; Eckstrand et al., 2008; Addington and Rapoport, 2009*, Kumra et al., 1998
	DUP Xp22.31 (342 Kb)	<13 y	−	not known	chrX:8,384,117-8,726,291	+	UCS	Walsh et al., 2008
	DEL Xp22.31 (1.68 Mb) STS	Male, 11 y	Congenital ichthyosis, microcencephalia, epilepsy. Language, motor, social, learning impairment, IQ 57, ADHD, ASD		*chrX:6,456,036-8,139,238*		UCS	Malik et al., 2017
	DUP Xq28 (6 a 35 Kb)	Female, 4 y	ASD, Epilepsy, Chiari 1	*de novo*	*chrX:15,29,55,334–15,29,61,664 to* *15,29,51,719–15,29,86,547*	*+*	UCS	Brownstein et al., 2016
	DEL Xq23-q28 (43 Mb)	Female, 12 y	ASD, poor motor coordination, IQ 67	*de novo*	−	+	P	Rudd et al., 2015
Y	DUP Yq11.221 (183 Kb)	Male, 8 y	Generalized anxiety disorder	*de novo*	chrY:14,441,161-14,623,937	+	UCS	Walsh et al., 2008

ADHD, Attention Deficit Hyperactivity Disorder; ASD, Autism Spectrum Disorder; CNV, Copy number variation; COPD, Chronic Obstructive Pulmonary Disease; DEL, Deletion; DUP, Duplication; iUPD, Uniparental isodisomy; ID, Intellectual disability; Kb, Kilobases; Mb, Mégabases; MDD, Major Depressive Disorder; MR, Mental Retardation; OCD, Obsessive compulsive disorder; P, Pathogenic; PDD-NOS, Pervasive developmental disorder not otherwise specified; UCS, Uncertain Clinical Significance; VCF, Velocardiofacial; Y, Years; * Literature review.

When available, phenotypes (sex, age of onset, other neurodevelopmental disorders and comorbidities) are described.

**Table 2:** Genomic microlesions (including single nucleotide variations) in COS patients with their localization, length, and type of inheritance.

Gene name	Gene localization	SNV ID number/mutation (protein level)	Population / Phenotypes	Inheritance	p-value	Bibliography
**FAMILY BASED ASSOCIATION STUDY**						
**DAOA (G72) / DAOA-AS1 (G30)**	13q33.2	rs1935058, rs3916967, rs2391191	n=64 (53 COS trios, 11 COS dyads)	−	0.015 to 0.5	Addington et al., 2004
**DTNBP1**	6p22.3	rs11558324	n=92 (73 COS + PDD-NOS trios, 19 COS + PDD-NOS dyads)	−	0.014	Gornick et al., 2005
**GAD1**	2q31.1	rs3749034, rs2270335, rs2241165	n=66 (55 COS + PDD-NOS trios, 11 COS + PDD-NOS dyads)	−	0.005	Addington et al., 2005
**NRG1**	8p12	rs35753505, rs2881272, rs327417	n=70 (59 COS + PDD-NOS trios, 11 COS + PDD-NOS dyads)	−	0.009 to 0.05	Addington et al., 2007
**CASE CONTROL STUDY**						
**BDNF**	11p13	val66met	65 patients (10.5 ± 3.7 y) vs 111 controls	−	0.03	Pakhomova et al., 2010
**COMT**	22q11.21	val158met	83 patients (<13 y) vs 208 controls	−	−	Raznahan et al., 2011
**TPH1**	11p15.3-p14	ala218cys	51 patients (<16 y) vs 148 controls	−	0.0058	Sekizawa et al., 2004
**GENES SEQUENCING (candidate genes or full exome)**						
**ATP1A3**	19q13.2	val129met	Male, onset: 6 y, motor delay	*de novo*	−	Smedemark-Margulies et al., 2016
		asp801asn	Male, onset: 10 y, ASD, dysmophia, motor, intellectual and learning delays. Recurent MDD		−	Chaumette et al., 2018
		glu815lys	Male, onset: 12 y, motor and communication impairments, dysmophia, ASD		−	
		ala813val	Male, onset: 10 y, ASD, motor and intellectual delays	mother	−	
**FXYD1**	19q13.12	arg90cys	Male, onset: 7 y, Asperger's disorder	inherited	−	
**FXYD6-FXYD2**	11q23.3	val101ala	Male, onset: 13 y		−	
**FXYD6**	11q23.3	gly73arg	Female, onset: 12 y		−	
**GPR153**	1p36.31	arg73cys	Male, onset: 12 y	*de novo*	−	Ambalavanan et al., 2016
**GTF2IRD1**	7q11.23	arg357cys	Female, onset: 12 y		−	
**ITGA6**	2q31.1	glu1063del	Female, onset: 12 y		−	
**LUZP4**	Xq23	arg278fs*10	−	mother	−	Ambalavanan et al., 2019
**OPHN1**	Xq12	met461val	IQ 88, PDDNOS		−	
**PCDH19**	Xq22.1	leu1022ile	−		−	
**RPS6KA3**	Xp22.12	arg723his	IQ 64, PDDNOS		−	
**RYR2**	1q43	glu746tyr	Male, onset: 8 y, PDDNOS, separation anxiety disorder, Asperger's disorder	*de novo*	−	Ambalavanan et al., 2016
**SEZ6**	17q11.2	thr229_thr231del	Male, onset: 11 y		−	
**TTBK1**	6p21.1	arg258gln	Male, onset: 7 y, Asperger's disorder		−	
**UPF3B**	Xq24	gln228fsX18	Male, 10 y, ADHD, PDDNOS, ASD	mother	−	Addington et al., 2011

ADHD, Attention Deficit Hyperactivity Disorder; ASD, Autism Spectrum Disorder; CNV, Copy number variation; COS, Childhood Onset Schizophrenia; DEL, Deletion; DUP, Duplication; MDD, Major Depressive Disorder; PDD-NOS, Pervasive developmental disorder not otherwise specified; SNV, Single Nucleotide Variation; Y, Years. When available, phenotypes (sex, age of onset, other neurodevelopmental disorders, and comorbidities) are described.

**Figure 1:**
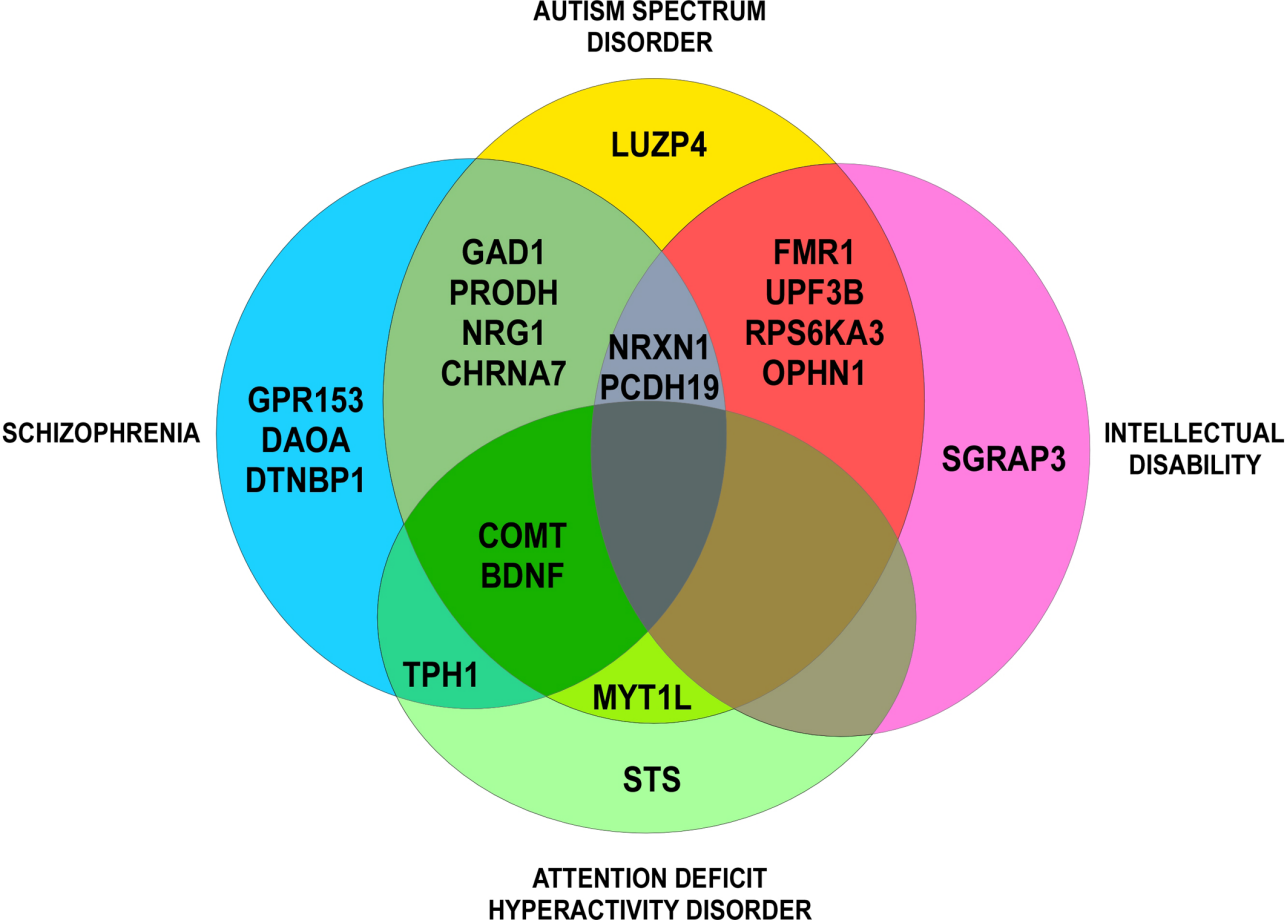
Venn diagram: evidence for genetic overlap in neurodevelopmental disorders (Phenocarta Database).

All CNVs were manually annotated using the University of California Santa Cruz (UCSC) Genome Browser (UCSC Mar. 2006 (NCBI36/hg18 or NCBI37/hg19) assembly; http://genome.ucsc.edu/). Regarding their type (gain or loss), their size, their genomic content, and making comparisons with external databases, we ranked each CNV as “pathogenic,” “uncertain clinical significance,” or “benign” (according to the American College of Medical Genetics standards and guidelines for interpretation and reporting of postnatal constitutional copy number variants). For each CNV, we checked on the Simons Foundation Autism Research Initiative (SFARI) Gene database (autism/genetic database, http://sfari.org) which CNV involved in COS was also involved in ASD. For each gene, we checked on the Phenocarta Database (https://gemma.msl.ubc.ca/home.html) the evidence linking genes to phenotypes of neurodevelopmental disorders (**Figure 1**, Venn diagram). Phenotypes were systematically described, if available.

**Figure 2:**
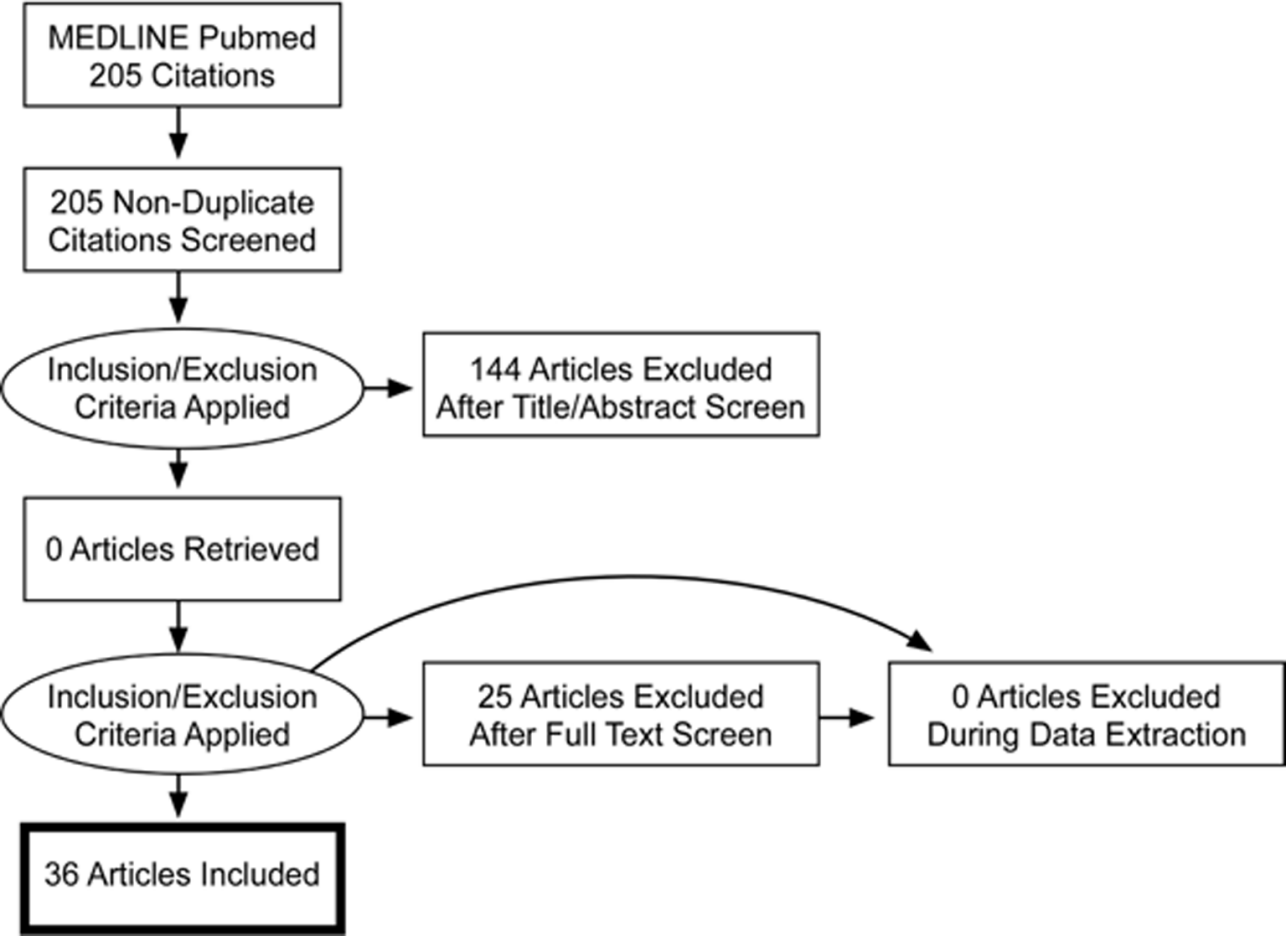
PRISMA Flowchart diagram.

The selection took place before September 2018. At this time, 36 articles (1994 to 2018) out of 205 (1982 to 2018) met the inclusion criteria. Article reviewing process, including selection and exclusion, is summarized in a PRISMA flow diagram (**Figure 2**). Two articles were added after the freezing of the inclusion process (41; 52). Mutations were identified in 21 chromosomes. The results were ranked either in ascending order of their chromosomal position for structural variants (cytogenetic abnormalities) (**Table 1**) or in alphabetical order according to their gene name for genetic variants (lesions at gene level) (**Table 2**).

## Results

### I Cytogenetic Abnormalities Associated With COS

**Figure 3:**
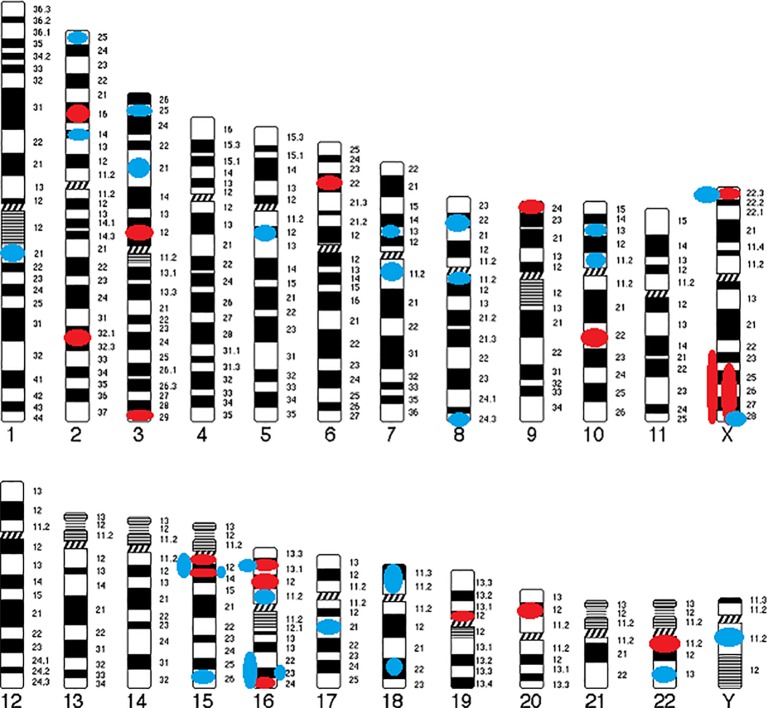
Genomic map of CNVs identified in COS. Circles indicate their position and color represents type of abnormality (Red circle, deletion; Blue circle, duplication).

The following cytogenetic abnormalities (n = 72, including 66 CNVs) were identified in 16 autosomes (1, 2, 3, 5, 6, 7, 8, 9, 10, 15, 16, 17, 18, 19, 20, and 22) and two sex chromosomes (X and Y) of 46 patients (17–40). The results were ranked in ascending order of their chromosomal position (**Table 1**) and were summarized in a genomic map (**Figure 3**).

1) CNVs
  - Deletions (CNVs) from 15Kb to 43Mb: 2p16.3, 2q31.2-q31.3, 3p12.2-p12.1, 3q29, 6p22.31, 9p24.2, 10q22.3, 15q11.2, 15q13.3, 16p12.1, 16p13.11, 16q24.1, 19p12, 20p12.1, 22q11.21, Xp22.31, Xq24-ter, and Xq23-q28;
  - Duplications (CNVs) from 120Kb to 17Mb: 1q21.3, 2p14, 2p25.3, 3p21.31, 3p25.3, 5q12.3, 7p13, 7q11.21-q11.22, 8p22, 8q11.23, 8q24.3, 10p11.23, 10p13, 15q11-q13, 15q13.3, 15q26.2–26.3, 16p11.2, 16p13.11, 16q22.2-ter, 16q22.3-q24.3, 16q23.3, 17q21.31, 18p11.31-p11.23, 18q22.1, 22q13.32, Xp22.31, Xq28, and Yq11.221.

Only six CNVs (9%) of our study are described in COS only: del2q31.2-q31.3 (smaller than the CNV described in ASD), del3p12.2-p12.1 (described in ASD as a duplication), delXq24-ter (larger than the CNV described in ASD), dup7p13 (described in ASD as a deletion), dup16q22.2-ter (larger than the CNV described in ASD), and dup16q22.3-q24.3 (larger than the CNV described in ASD).

In an on-site case series and literature review focusing on Childhood-Onset Schizophrenia Spectrum Disorders (SSDs; larger spectrum than COS), Giannitelli et al. (2018) showed that some CNVs, previously un-described in COS patients, are associated to childhood psychosis: 1q21.1 deletion, 1q21.1 duplication, Williams-Beuren region (7q11.23) duplication and 16p11.2 deletion (Giannitelli et al., 2018).

The phenotypes of only 15 out of the 46 patients were fully described (33%). The neurodevelopmental disorders that have been presented are: motor impairments (fine or growth milestones delay, coordination disability, or tics) in 11/15 patients, language retardation in 7/15 patients, intellectual disability (IQ < 70) in two patients, and ASD in five patients [(including 1 Pervasive Developmental Disorder-Not Otherwise Specified (PDD-NOS)]. Social impairment was present in six other patients with COS and ADHD in three patients. Inattention impairment was specified in only one patient. The psychiatric comorbidities that have been highlighted are: two cases with mood disorders (major depressive disorder or dysthymia) and two cases with anxiety disorders. The somatic comorbidities detected are: dysmorphia in four cases and epilepsy in two cases. Sporadic cases of hypospadias, ureteric reflux, congenital ichthyosis, Chiari type 1, or celiac disease were also described.

2) Other DNA Lesions
  - Aneuploidy
    One case of Trisomy X (47XXX) and two cases of Turner syndrome (45X0), one atypical form (46,X,del(X)(q24-ter) and one with mosaicism 46,X,i(X)(q10)(22%)/45,X(78%) (Eckstrand et al., 2008; Addington and Rapoport, 2009);
  - Uniparental and Segmental isoDisomy (iUSD)
    In isodisomy, both copies of a chromosomal set are inherited from one parent (the mother or the father). An iUSD on 5q32-ter (35Mb) was described in a patient with COS (Eckstrand et al., 2008; Addington and Rapoport, 2009; Seal et al., 2006);
  - Translocation 1, 7: p22q22 (Gordon et al., 1994; Nicolson et al., 1999; Yan et al., 2000; Eckstrand et al., 2008; Idol et al., 2008; Addington and Rapoport, 2009)
  - Trinucleotide Repeat Expansions
    - CGG Expansions: Although a link between Fragile X syndrome (FXS) and COS has never been reported, Vantalon et al. (2005) described a 1.5 Kb expansion and complete methylation of the CpG island upstream of FMR1 in a 9 years-old girl with COS, dysmorphia, learning and social impairments, and mild mental retardation (Vantalon et al., 2005). This abnormality is inherited from the mother who carries an FXS premutation. Interestingly, instead of being unaffected or weakly affected as most patients carrying a premutation, the mother presents schizotypal personality. It seems that the severity of the schizophrenic spectrum disorder, which affects both mother and daughter with gradual severity, is linked to the CGG expansion degree. Effect of the hazard could not be excluded in this case (Vantalon et al., 2005).
    - CAG/CTG Expansion: a longer repeat expansion on chromosome 18 was found in a COS cohort and in a male subclass, with a significant p-value especially for the males (0.036 and 0.002, respectively; Wilcoxon–Mann–Whitney U test) (Burgess et al., 1998).
  - Genetic Syndromes
    In their recent review, Giannitelli et al. (2018) showed that some genetic syndromes, previously un-described in COS, are associated to childhood-onset SSDs: juvenile Huntington disease, Prader-Willi syndrome, Steinert myotonia, Ondine syndrome, Rubinstein-Taybi syndrome, and GLUT1 deficiency syndrome (Giannitelli et al., 2018).

### II Genes Associated With COS

In total, 32 candidate genes have been described on 12 autosomes (1, 2, 3, 6, 7, 8, 11, 13, 15, 17, 19, and 22) and 1 sex chromosome (X) (**Tables 1** and **2**) through the following studies (Addington et al., 2004; Sekizawa et al., 2004; Gornick et al., 2005; Addington et al., 2005; Addington et al., 2007; Pakhomova et al., 2010; Addington et al., 2011; Raznahan et al., 2011; Smedemark-Margulies et al., 2016; Chaumette et al., 2018; Ambalavanan et al., 2019):

1) Genetic Association Studies (**Table 2**)
  - Family-Based “Transmission Disequilibrium Test” Studies (From 2004 to 2007)
    Transmission disequilibrium test, a family-based association test for the presence of genetic linkage between a genetic marker and a trait, was used to describe the following candidate genes: *DAOA, DAOA-AS1, DTNBP1, GAD1*, and *NRG1*. The Linkage Disequilibrium Analyses for Quantitative and Discrete Traits (QTDT) program was used to make statistical analysis (p-values).
    - *DAOA* and *DAOA-AS1* (Addington et al., 2004): three Single Nucleotide Polymorphisms (SNPs) are associated with COS (rs1935058, rs3916967, and rs2391191 (p = 0.5, 0.015, 0.3, respectively)). The most significant SNP (p = 0.015) is rs3916967 (genomic sequence reference: NG_012694.1:g.4133T > C);
    - *DTNBP1* (Gornick et al., 2005): one SNP, rs11558324 (NG_009309.1:g.5154A > G), is associated with COS (p = 0.014) and two two-marker haplotypes (containing rs11558324) are also associated with COS (p = 0.021, 0.008),
    - *GAD 1* (Addington et al., 2005): three four-marker haplotypes are associated with COS (p = 0.005);
    - *NRG1* (Addington et al., 2007): individual markers show association with COS (rs35753505, rs2881272, 420M9-1395 (microsatellite), and rs327417, with p-value between 0.009 and 0.05). The most significant SNP (p = 0.009) is rs327417 (NG_012005.2:g.341913G > A). Further, several novel four-marker haplotypes are associated with COS (lowest p = 0.0004).
  - Population-Based “Case vs. Control” Studies (From 2004 to 2011)
    A polymorphism (VAL66MET) in the *BDNF* (11p13) gene was associated with COS in a 65 patient cohort (10.5 ± 3.7 years old at onset) vs. 111 controls (p = 0.03; χ² test) (Pakhomova et al., 2010). A mutation (VAL158MET) in *COMT* (22q11.21) that increases protein activity levels in the brain accelerated adolescent cortical thinning (MRI findings) in both schizophrenia probands and their siblings (with resolution after a certain age for siblings), illustrating the influence of dopaminergic disruption on brain cortical maturation. Authors analyzed data from an 83 COS patient cohort vs. 208 healthy controls (Raznahan et al., 2011). A mutation (A218C) in the *TPH1* (11p15.3-p14) gene (p = 0.0058; χ² test) is described in 51 patients (early adolescent onset cohort before 16 years old) vs. 148 controls (Sekizawa et al., 2004).
2) Gene Sequencing Studies (2011 to Present)
  Finally, five studies by DNA sequencing (candidate genes or whole exome sequencing) have allowed identifying the following 18 mutations in 11 genes (Addington et al., 2011; Ambalavanan et al., 2016; Smedemark-Margulies et al., 2016; Chaumette et al., 2018; Ambalavanan et al., 2019).

- Six SNVs inherited from a healthy mother:
  - Five X-linked recessive variants: *LUZP4* (arg278fs*10), *OPHN1* (met461val), *PCDH19* (leu1022ile), *RPS6KA3* (arg723his), and *UPF3B* gene (gln228fsX18) that also segregates in the sibling.
  - One SNV (ala813val) in *ATP1A3* (a boy with onset at 10 years old and co-morbid ASD).
- Three SNVs inherited (missenses variants) in the FXYD gene family. *FXYD1* (arg90cys), *FXYD6-FXYD2* (val101ala), and *FXYD6* (gly73arg) genes. Only the patient with the mutation in FXYD1 presents a co-morbid ASD (Asperger’s disorder).
- Nine *de novo* SNVs in the following genes: *ATP1A3* (val129met; asp801asn; glu815lys), *GPR153* (arg73cys), *GTF2IRD1* (arg357cys), *ITGA6* (glu1063del), *RYR2* (glu746tyr), *SEZ6* (thr229_thr231del), and *TTBK1* (arg258gln).

The ATPase Na^+^/K^+^ transporting Alpha-3 Polypeptide (*ATP1A3* gene) encodes the alpha-3 catalytic subunit of the Na^+^/K^+^-ATPase transmembrane ion pump mapping at 19q12-q13.2 (Harley et al., 1988). The *ATP1A3* isoform is exclusively expressed in neurons of various brain regions, including the basal ganglia, hippocampus, and cerebellum (summary by Rosewich et al., 2012). Mutations in this gene have been associated with a spectrum of disorders depending on the domain they affect in the corresponding protein. The majority of mutations associated with rapid-onset dystonia parkinsonism, or dystonia-12 (*DYT12*), were located in exons 8 and 14 whereas those with alternating hemiplegia of childhood-2 (AHC2) were located in exons 17 and 18 and in general they seem to affect transmembrane and functional domains, being the most severe dysfunctions. By genetic analysis of clinical data from 155 patients with AHC2, 132 confirmed to have *ATP1A3* mutations. Among those with AHC2, the most frequent mutations were D801N (in 43%), E815K (in 16%) and G947R (in 11%). E815K was associated with a severe phenotype, with greater intellectual and motor disability; D801N appeared to confer a milder phenotype and G947R correlated with the most favorable prognosis. For those with epilepsy, the age at seizure onset was earlier for patients with the E815K or G947R mutations than for those with the D801N mutation (Panagiotakaki et al., 2015). In 10 patients from three unrelated families with cerebellar ataxia, areflexia, pes cavus, optic atrophy, and sensorineural hearing loss (CAPOS; 601338) (Demos et al., 2014), the same heterozygous missense mutation in the *ATP1A3* gene was identified (E818K; OMIM 182350.0014).

The G Protein-coupled Receptor 153 (*GPR153*) gene, located on 1p36.31, belongs to the large rhodopsin (RHO; OMIM 180380) family of GPCRs (Gloriam et al., 2005) and shows a highest similarity to serotonin receptors, (Gloriam et al., 2005). Furthermore, knockdown of *GPR153* in mice showed reduction in food intake and increased anxiety according to the elevated plus Maze test (Sreedharan et al., 2011).

The InTeGrin Alpha-6 (*ITGA6*) gene is located on 2q31.1 (Hogervorst et al., 1991). While functional absence of *ITGA6* has been associated with epidermolysis bullosa (Hogervorst et al., 1991; Georges-Labouesse et al., 1996), a few works addressed the role of *ITGA6* in neurons. Alpha-6 integrin was initially reported to be involved in neural migration (Yao et al., 2018). In addition, recent data suggested that α6 and β1 integrins may play a role in mediating Schwann cell interactions with axons and promote axonal regeneration (Chang et al., 2018).

The RYanodine Receptor 2 (*RYR2*) gene—located on chromosome 1 between q42.1 et q43—encodes a calcium channel that is located in the sarcoplasmic reticulum and is the major source of calcium required for cardiac muscle excitation-contraction coupling. (Bhuiyan et al., 2007). Ryr2-/- mice die at approximately embryonic day 10 with morphologic abnormalities in the heart tube. Ca^2+^ signaling has been associated with ASD (Kabir et al., 2016; Stephenson et al., 2017; Castagnola et al., 2018) and with other psychiatric and neurological diseases (Heyes et al., 2015). It is not surprising that *RYR2* was linked to ASD by genetic studies (Lu and Cantor, 2012; Soueid et al., 2016; Chen et al., 2017). However, it is very interesting to underline that an SNP in this gene was associated with ASD in families with only affected males in contrast with those with affected females (Lu and Cantor, 2012) suggesting that *RYR2* is a sex-related genetic factor for ASD.

The SEiZure-related 6 (*SEZ6*) gene is located on the 17q11.2 chromosome. Sez6 types 1 and 2 have an N-terminal signal sequence, followed by a threonine-rich region, a Short Consensus Repeat (SCR), a CUB-like domain, a second SCR, a second CUB-like domain, three tandem SCRs, a transmembrane domain, and a cytoplasmic C-terminal tail. They differ only in the region between the last SCR and the transmembrane domain. *SEZ6* was predicted to be involved in neuronal maturation and plasticity (Miyazaki et al., 2006). Recently mutations and altered expression of this gene have been associated with Alzheimer’s and Niemann-Pick disease (Causevic et al., 2018; Paracchini et al., 2018).

The Tau TuBulin Kinase 2 (*TTBK2*) gene, located on 15q15.2, encodes a member of the casein kinase (CK1) group of eukaryotic protein kinases. *TTBK1* has been implicated in Alzheimer’s disease (OMIM 104300) and in neurofibrillary tangles formation (Sato et al., 2006). Mutations in this gene also cause spinocerebellar ataxia 11 (SCA11; 604432). SCA11 is a pure progressive cerebellar ataxia that has been linked to 15q14-q21 (Worth et al., 1999; Houlden et al., 2007). In an 8-generation English family they found a one-base insertion in the *TTBK2* gene creating a premature stop codon and a truncation of the normal protein (OMIM 611695.0001). In a second family of Pakistani ancestry, a different mutation was found (OMIM 611695.0002). Goetz et al. (2012) concluded that *TTBK2* is required for removal of CP110 for the initiation of ciliogenesis (Goetz et al., 2012).

3) Candidate Genes From Cytogenetic Studies (**Table 1**)

Interesting candidate genes deleted, duplicated, or truncated by the CNVs have also been found in cytogenetic studies (see above). These genes have brain expression and are mostly described in other neurodevelopmental or psychiatric disorders (**Figure 1**). Nine genes are described as putative COS-causing genes: *CHRNA7* (15q13.3), *DGCR6* (22q11.2), *FMR1* (Xq27.3), *MYT1L* (2p25.3), *NRXN1* (2p16.3), *PRODH* (22q11.2), *PTK2* (8q24.3), *STS* (Xp22.31), and *SRGAP3* (3p25.3) (Yan et al., 1998; Nicolson et al., 1999; Usiskin et al., 1999; Liu et al., 2002; Sporn et al., 2004; Vantalon et al., 2005; Eckstrand et al., 2008; Walsh et al., 2008; Addington and Rapoport, 2009; Rapoport et al., 2009; Wilson et al., 2011; Lee et al., 2012; Ahn et al., 2014; Duong et al., 2015; Zhou et al., 2016; Malik et al., 2017).

## Conclusions

COS is a neurodevelopmental disorder with several degrees of complexity (clinical and genetic heterogeneity). Clinically, getting the diagnostic is very challenging (severe disorder, comorbidities, and association with other neurodevelopmental disorders) (Gochman et al., 2011). The clinical overlap with ASD is well documented and in our study we found a co-morbidity rate (33%) nearly equal to the National Institute of Mental Health (NIMH) COS cohort rate (28%) (Rapoport et al., 2009). The genetic overlap with ASD is also well documented and we show that 91% of described CNVs are also described in ASD (*SFARI*). In the literature, we found only 20% of COS patients with co-morbid ADHD vs. 84% according to Ross et al. and we hypothesize that this trouble was under-diagnosed in schizophrenia studies (Ross et al., 2006). All intellectual, motor, communication, and learning impairments are also frequently observed in COS (Ross et al., 2006; Nicolson et al., 1999). Psychiatric comorbidities were rarely described (two cases of mood disorders and two cases of anxiety disorders), which was an unexpected outcome given the published literature (Ross et al., 2006). Here, we highlight that only one-third of the full phenotypes associated with the mutations published in the literature are described, which constitutes a significant loss of information for researchers. Therefore, it appears fundamental to carry out preliminary work before genetic testing: perform a rigorous and homogeneous phenotypic characterization using International Classification of Disease (*ICD-10* and *DSM-5*) with standardized and internationally validated psychiatric categorical assessments and in accordance with medical history (including perinatology), biography (with significant life event and trauma), and environmental factors (such as toxic exposure).

COS is characterized by a complex genetic architecture with both inherited and *de novo* mutations distributed in almost all chromosomes. Most of the genes causing COS are unknown yet. It is interesting that, the few that have been already proposed (see before) are involved both in neurodevelopmental and neurodegenerative disorders such as Parkinson, Alzheimer, or ataxia. Moreover, schizophrenia has been shown to have complex genetic traits with high polygenic risk (Ahn et al., 2016). Thus, a second hit (or more), in addition to CNV, is probably essential to explain the phenotypes. It includes *de novo* SNVs, other CNVs and/or environmental factors (e.g., trauma at early childhood, central nervous system infections or injuries) (Davis et al., 2016). At the interplay between genetic and environmental factors, epigenetics opens new perspectives to understand biological mechanisms of psychosis. In fact, recent findings suggest that pangenomic methylation changes during adolescence accompany conversion to psychosis (Kebir et al., 2018). In clinical practice, as suggested by Szego et al. for ASD (Szego and Zawati, 2016), it would seem useful to propose to COS patients genetic sequencing instead or in addition to microarrays (Anagnostou et al., 2014; Soden et al., 2014) to improve genetic testing and to allow *de novo* SNV detection.

In research, the major challenge of the upcoming years will be the analysis of big data from NGS (prioritization and interpretation of DNA variations) (Richards et al., 2015) and the experimental validation of putative mutations. Sharing data with other teams around the world will be helpful to unravel the molecular pathology of COS and its underlying causes, paving the way for an early therapeutic intervention.

## Author Contributions

AF, FA, and BB: contributed to the conceptualization of the study and drafted the first version of the manuscript. All other authors MD, ST, ED, and MC have revised first version of the manuscript critically for important intellectual content and approved the final version.

## Funding

This study was supported by INSERM, CNRS, Université Côte d’Azur and Hôpitaux pédiatriques de Nice CHU-Lenval; ANR-11-LABX-0028-01 and ANR-15-CE16-0015 to BB; Monaco Against Autism (MONAA) Foundation to AF, MC, FA, and BB. MD is recipient of a Signalife-LabEx Program international Ph.D.

## Conflict of Interest

The authors declare that the research was conducted in the absence of any commercial or financial relationships that could be construed as a potential conflict of interest.

## References

[B1] Addington A. M. Rapoport J. L. (2009). The genetics of childhood-onset schizophrenia: when madness strikes the prepubescent. Curr. Psychiatry Rep. 11 (2), 156–161. 19302770 10.1007/s11920-009-0024-y PMC2763299

[B2] Addington A. M. Gornick M. Sporn A. L. Gogtay N. Greenstein D. Lenane M. (2004). Polymorphisms in the 13q33.2 gene G72/G30 are associated with childhood-onset schizophrenia and psychosis not otherwise specified. Biol. Psychiatry 55 (10), 976–980. 10.1016/j.biopsych.2004.01.024 15121480

[B3] Addington A. M. Gornick M. Duckworth J. Sporn A. Gogtay N. Bobb A. (2005). GAD1 (2q31.1), which encodes glutamic acid decarboxylase (GAD67), is associated with childhood-onset schizophrenia and cortical gray matter volume loss. Mol. Psychiatry 10 (6), 581–588. 10.1038/sj.mp.4001599. 15505639

[B4] Addington A. M. Gornick M. C. Shaw P. Seal J. Gogtay N. Greenstein D. (2007). Neuregulin 1 (8p12) and childhood-onset schizophrenia: susceptibility haplotypes for diagnosis and brain developmental trajectories. Mol. Psychiatry 12 (2), 195–205. 10.1038/sj.mp.4001906 17033632

[B5] Addington A. M. Gauthier J. Piton A. Hamdan F. F. Raymond A. Gogtay N. (2011). A novel frameshift mutation in UPF3B identified in brothers affected with childhood onset schizophrenia and autism spectrum disorders. Mol. Psychiatry 16 (3), 238–239. 10.1038/mp.2010.59 20479756 PMC3024438

[B6] Ahn K. Gotay N. Andersen T. M. Anvari A. A. Gochman P. Lee Y. (2014). High rate of disease-related copy number variations in childhood onset schizophrenia. Mol. Psychiatry 19 (5), 568–572. 10.1038/mp.2013.59 23689535 PMC5157161

[B7] Ahn K. An S. S. Shugart Y. Y. Rapoport J. L. (2016). Common polygenic variation and risk for childhood-onset schizophrenia. Mol. Psychiatry 21 (1), 94–96. 10.1038/mp.2014.158 25510512

[B8] Ambalavanan A. Girard S. L. Ahn K. Zhou S. Dionne-Laporte A. Spiegelman D. (2016). De novo variants in sporadic cases of childhood onset schizophrenia. Eur. J. Hum. Genet. 24 (6), 944–948. 10.1038/ejhg.2015.218 26508570 PMC4867457

[B9] Ambalavanan A. Chaumette B. Zhou S. Xie P. He Q. Spiegelman D. (2019). Exome sequencing of sporadic childhood-onset schizophrenia suggests the contribution of X-linked genes in males. Am. J. Med. Genet. B Neuropsychiatr. Genet. 180 (6), 335–340. 10.1002/ajmg.b.32683 30378261

[B10] American Psychiatric Association (2013). Diagnostic and statistical manual of mental disorders (DSM-5). Washington, DC: American Psychiatric Pub.

[B11] Anagnostou E. Zwaigenbaum L. Szatmari P. Fombonne E. Fernandez B. A. Woodbury-Smith M. (2014). Autism spectrum disorder: advances in evidence-based practice. CMAJ 186 (7), 509–519. 10.1503/cmaj.121756 24418986 PMC3986314

[B12] Asarnow R. F. Forsyth J. K. (2013). Genetics of childhood-onset schizophrenia. Child Adolesc. Psychiatr. Clin. N Am. 22 (4), 675–687. 10.1016/j.chc.2013.06.004 24012080 PMC4364758

[B13] Bhuiyan Z. A. van den Berg M. P. van Tintelen J. P. Bink-Boelkens M. T. Wiesfeld A. C. Alders M. (2007). Expanding spectrum of human RYR2-related disease: new electrocardiographic, structural, and genetic features. Circ. 116 (14), 1569–1576. 10.1161/CIRCULATIONAHA.107.711606. 17875969

[B14] Biswas P. Malhotra S. Malhotra A. Gupta N. (2006). Comparative study of neuropsychological correlates in schizophrenia with onset in childhood, adolescence and adulthood. Eur. Child Adolesc. Psychiatry 15 (6), 360–366. 10.1007/s00787-006-0542-7 16604435

[B15] Brownstein C. A. Kleiman R. J. Engle E. C. Towne M. C. D’Angelo E. J. Yu T. W. (2016). Overlapping 16p13.11 deletion and gain of copies variations associated with childhood onset psychosis include genes with mechanistic implications for autism associated pathways: two case reports. Am. J. Med. Genet. A 170A (5), 1165–1173. 10.1002/ajmg.a.37595 26887912 PMC4833544

[B16] Burd L. Kerbeshian J. (1987). A North Dakota prevalence study of schizophrenia presenting in childhood. J. Am. Acad. Child Adolesc. Psychiatry 26 (3), 347–350. 10.1097/00004583-198705000-00012 3496327

[B17] Burgess C. E. Lindblad K. Sidransky E. Yuan Q. P. Long R. T. Breschel T. (1998). Large CAG/CTG repeats are associated with childhood-onset schizophrenia. Mol. Psychiatry 3 (4), 321–327. 10.1038/sj.mp.4000405 9702740

[B18] Castagnola S. Delhaye S. Folci A. Paquet A. Brau F. Duprat F. (2018). New Insights Into the Role of Cav2 Protein Family in Calcium Flux Deregulation in Fmr1-KO Neurons. Front. Mol. Neurosci. 11, 342. 10.3389/fnmol.2018.00342 30319351 PMC6170614

[B19] Causevic M. Dominko K. Malnar M. Vidatic L. Cermak S. Pigoni M. (2018). BACE1-cleavage of Sez6 and Sez6L is elevated in Niemann-Pick type C disease mouse brains. PloS One 13 (7), e0200344. 10.1371/journal.pone.0200344 29979789 PMC6034874

[B20] Chang I. A. Kim K. J. Namgung U. (2018). alpha6 and beta1 Integrin heterodimer mediates schwann cell interactions with axons and facilitates axonal regeneration after peripheral nerve injury. Neurosci. 371, 49–59. 10.1016/j.neuroscience.2017.11.046 29223350

[B21] Chaumette B. Ferrafiat V. Ambalavanan A. Goldenberg A. Dionne-Laporte A. Spiegelman D. (2018). Missense variants in ATP1A3 and FXYD gene family are associated with childhood-onset schizophrenia. Mol. Psychiatry. 10.1038/s41380-018-0103-8 PMC6291354 29895895

[B22] Chen C. Van Horn J. D. Consortium G. R. (2017). Developmental neurogenetics and multimodal neuroimaging of sex differences in autism. Brain Imaging Behav. 11 (1), 38–61. 10.1007/s11682-015-9504-3. 26781567 PMC4955643

[B23] Davis J. Eyre H. Jacka F. N. Dodd S. Dean O. McEwen S. (2016). A review of vulnerability and risks for schizophrenia: beyond the two hit hypothesis. Neurosci. Biobehav. Rev. 65, 185–194. 10.1016/j.neubiorev.2016.03.017 27073049 PMC4876729

[B24] Demos M. K. van Karnebeek C. D. Ross C. J. Adam S. Shen Y. Zhan S. H. (2014). A novel recurrent mutation in ATP1A3 causes CAPOS syndrome. Orphanet J. Rare Dis. 9, 15. 10.1186/1750-1172-9-15 24468074 PMC3937150

[B25] Duong L. T. Hoeffding L. K. Petersen K. B. Knudsen C. D. Thygesen J. H. Klitten L. L. (2015). Two rare deletions upstream of the NRXN1 gene (2p16.3) affecting the non-coding mRNA AK127244 segregate with diverse psychopathological phenotypes in a family. Eur. J. Med. Genet. 58 (12), 650–653. 10.1016/j.ejmg.2015.11.004 26563496

[B26] Eckstrand K. Addington A. M. Stromberg T. Merriman B. Miller R. Gochman P. (2008). Sex chromosome anomalies in childhood onset schizophrenia: an update. Mol. Psychiatry 13 (10), 910–911. 10.1038/mp.2008.67 18800051 PMC4316819

[B27] Georges-Labouesse E. Messaddeq N. Yehia G. Cadalbert L. Dierich A. Le Meur M. (1996). Absence of integrin alpha 6 leads to epidermolysis bullosa and neonatal death in mice. Nat. Genet. 13 (3), 370–373. 10.1038/ng0796-370 8673141

[B28] Giannitelli M. Consoli A. Raffin M. Jardri R. Levinson D. F. Cohen D. (2018). An overview of medical risk factors for childhood psychosis: Implications for research and treatment. Schizophr. Res. 192, 39–49. 10.1016/j.schres.2017.05.011 28526280

[B29] Gloriam D. E. Schioth H. B. Fredriksson R. (2005). Nine new human Rhodopsin family G-protein coupled receptors: identification, sequence characterization and evolutionary relationship. Biochim. Biophys. Acta 1722 (3), 235–246. 10.1016/j.bbagen.2004.12.001 15777626

[B30] Gochman P. Miller R. Rapoport J. L. (2011). Childhood-onset schizophrenia: the challenge of diagnosis. Curr. Psychiatry Rep. 13 (5), 321–322. 10.1007/s11920-011-0212-4 21713647 PMC3289250

[B31] Goetz S. C. Liem K. F. Jr. Anderson K. V. (2012). The spinocerebellar ataxia-associated gene Tau tubulin kinase 2 controls the initiation of ciliogenesis. Cell 151 (4), 847–858. 10.1016/j.cell.2012.10.010 23141541 PMC3496184

[B32] Gordon C. T. Krasnewich D. White B. Lenane M. Rapoport J. L. (1994). Brief report: translocation involving chromosomes 1 and 7 in a boy with childhood-onset schizophrenia. J. Autism Dev. Disord. 24 (4), 537–545. 10.1007/bf02172134 7961336

[B33] Gornick M. C. Addington A. M. Sporn A. Gogtay N. Greenstein D. Lenane M. (2005). Dysbindin (DTNBP1, 6p22.3) is associated with childhood-onset psychosis and endophenotypes measured by the Premorbid Adjustment Scale (PAS). J. Autism Dev. Disord. 35 (6), 831–838. 10.1007/s10803-005-0028-3 16283082

[B34] Harley H. G. Brook J. D. Jackson C. L. Glaser T. Walsh K. V. Sarfarazi M. (1988). Localization of a human Na+,K+-ATPase alpha subunit gene to chromosome 19q12—q13.2 and linkage to the myotonic dystrophy locus. Genomics 3 (4), 380–384. 10.1016/0888-7543(88)90131-0 2907504

[B35] Heyes S. Pratt W. S. Rees E. Dahimene S. Ferron L. Owen M. J. (2015). Genetic disruption of voltage-gated calcium channels in psychiatric and neurological disorders. Prog. Neurobiol. 134, 36–54. 10.1016/j.pneurobio.2015.09.002. 26386135 PMC4658333

[B36] Hogervorst F. Kuikman I. van Kessel A. G. Sonnenberg A. (1991). Molecular cloning of the human alpha 6 integrin subunit. Alternative splicing of alpha 6 mRNA and chromosomal localization of the alpha 6 and beta 4 genes. Eur. J. Biochem. 199 (2), 425–433. 10.1111/j.1432-1033.1991.tb16140.x 2070796

[B37] Houlden H. Johnson J. Gardner-Thorpe C. Lashley T. Hernandez D. Worth P. (2007). Mutations in TTBK2, encoding a kinase implicated in tau phosphorylation, segregate with spinocerebellar ataxia type 11. Nat. Genet. 39 (12), 1434–1436. 10.1038/ng.2007.43 18037885

[B38] Idol J. R. Addington A. M. Long R. T. Rapoport J. L. Green E. D. (2008). Sequencing and analyzing the t(1;7) reciprocal translocation breakpoints associated with a case of childhood-onset schizophrenia/autistic disorder. J. Autism Dev. Disord. 38 (4), 668–677. 10.1007/s10803-007-0435-8 17879154

[B39] Kabir Z. D. Lee A. S. Rajadhyaksha A. M. (2016). L-type Ca(2+) channels in mood, cognition and addiction: integrating human and rodent studies with a focus on behavioural endophenotypes. J. Physiol. 594 (20), 5823–5837. 10.1113/JP270673. 26913808 PMC5063939

[B40] Kallmann F. J. Roth B. (1956). Genetic aspects of preadolescent schizophrenia. Am. J. Psychiatry 112 (8), 599–606. 10.1176/ajp.112.8.599 13292546

[B41] Kebir O. Chaumette B. Krebs M. O. (2018). Epigenetic variability in conversion to psychosis: novel findings from an innovative longitudinal methylomic analysis. Transl. Psychiatry 8 (1), 93. 10.1038/s41398-018-0138-2 29695761 PMC5916914

[B42] Kumra S. Wiggs E. Krasnewich D. Meck J. Smith A. C. Bedwell J. (1998). Brief report: association of sex chromosome anomalies with childhood-onset psychotic disorders. J. Am. Acad. Child Adolesc. Psychiatry 37 (3), 292–296. 10.1097/00004583-199803000-00014 9519634

[B43] Lee Y. Mattai A. Long R. Rapoport J. L. Gogtay N. Addington A. M. (2012). Microduplications disrupting the MYT1L gene (2p25.3) are associated with schizophrenia. Psychiatr. Genet. 22 (4), 206–209. 10.1097/YPG.0b013e328353ae3d 22547139 PMC3384746

[B44] Liu H. Heath S. C. Sobin C. Roos J. L. Galke B. L. Blundell M. L. (2002). Genetic variation at the 22q11 PRODH2/DGCR6 locus presents an unusual pattern and increases susceptibility to schizophrenia. Proc. Natl. Acad. Sci. U S A 99 (6), 3717–3722. 10.1073/pnas.042700699 11891283 PMC122590

[B45] Lu A. T. Cantor R. M. (2012). Allowing for sex differences increases power in a GWAS of multiplex Autism families. Mol. Psychiatry 17 (2), 215–222. 10.1038/mp.2010.127. 21151189

[B46] Maier W. (1999). Diagnostic classification of psychiatric disorders and familial-genetic research. Dialogues Clin. Neurosci. 1 (3), 191–196. 22033840 10.31887/DCNS.1999.1.3/wmaier PMC3181578

[B47] Malik A. Amer A. B. Salama M. Haddad B. Alrifai M. T. Balwi M. A. (2017). X-linked ichthyosis associated with psychosis and behavioral abnormalities: a case report. J. Med. Case Rep. 11 (1), 267. 10.1186/s13256-017-1420-2 28934990 PMC5609014

[B48] Miyazaki T. Hashimoto K. Uda A. Sakagami H. Nakamura Y. Saito S. Y. (2006). Disturbance of cerebellar synaptic maturation in mutant mice lacking BSRPs, a novel brain-specific receptor-like protein family. FEBS Lett. 580 (17), 4057–4064. 10.1016/j.febslet.2006.06.043 16814779

[B49] Nicolson R. Rapoport J. L. (1999). Childhood-onset schizophrenia: rare but worth studying. Biol. Psychiatry 46 (10), 1418–1428. 10.1016/s0006-3223(99)00231-0 10578456

[B50] Nicolson R. Giedd J. N. Lenane M. Hamburger S. Singaracharlu S. Bedwell J. (1999). Clinical and neurobiological correlates of cytogenetic abnormalities in childhood-onset schizophrenia. Am. J. Psychiatry 156 (10), 1575–1579. 10.1176/ajp.156.10.1575 10518169

[B51] Pakhomova S. A. Korovaitseva G. I. Monchakovskaia M. Vil’ianov V. B. Frolova L. P. Kasparov S. V. (2010). [Molecular-genetic study of early-onset schizophrenia]. Zh Nevrol. Psikhiatr. Im. S S Korsakova 110 (2), 66–69. 20436453

[B52] Panagiotakaki E. De Grandis E. Stagnaro M. Heinzen E. L. Fons C. Sisodiya S. (2015). Clinical profile of patients with ATP1A3 mutations in Alternating Hemiplegia of Childhood-a study of 155 patients. Orphanet J. Rare Dis. 10, 123. 10.1186/s13023-015-0335-5 26410222 PMC4583741

[B53] Paracchini L. Beltrame L. Boeri L. Fusco F. Caffarra P. Marchini S. (2018). Exome sequencing in an Italian family with Alzheimer’s disease points to a role for seizure-related gene 6 (SEZ6) rare variant R615H. Alzheimers Res. Ther. 10 (1), 106. 10.1186/s13195-018-0435-2 30309378 PMC6182820

[B54] Petty L. K. Ornitz E. M. Michelman J. D. Zimmerman E. G. (1984). Autistic children who become schizophrenic. Arch. Gen. Psychiatry 41 (2), 129–135. 10.1001/archpsyc.1984.01790130023003 6696593

[B55] Rapoport J. Chavez A. Greenstein D. Addington A. Gogtay N. (2009). Autism spectrum disorders and childhood-onset schizophrenia: clinical and biological contributions to a relation revisited. J. Am. Acad. Child Adolesc. Psychiatry 48 (1), 10–18. 10.1097/CHI.0b013e31818b1c63 19218893 PMC2664646

[B56] Rapoport J. Chavez A. Greenstein D. Addington A. Gogtay N. (2009). Autism spectrum disorders and childhood-onset schizophrenia: clinical and biological contributions to a relation revisited. J. Am. Acad. Child Adolesc. Psychiatry 48 (1), 10–18. 10.1097/CHI.0b013e31818b1c63 19218893 PMC2664646

[B57] Rapoport J. L. Giedd J. N. Gogtay N. (2012). Neurodevelopmental model of schizophrenia: update 2012. Mol. Psychiatry 17 (12), 1228–1238. 10.1038/mp.2012.23 22488257 PMC3504171

[B58] Raznahan A. Greenstein D. Lee Y. Long R. Clasen L. Gochman P. (2011). Catechol-o-methyl transferase (COMT) val158met polymorphism and adolescent cortical development in patients with childhood-onset schizophrenia, their non-psychotic siblings, and healthy controls. NeuroImage 57 (4), 1517–1523. 10.1016/j.neuroimage.2011.05.032 21620981 PMC3285479

[B59] Richards S. Aziz N. Bale S. Bick D. Das S. Gastier-Foster J. (2015). Standards and guidelines for the interpretation of sequence variants: a joint consensus recommendation of the American College of Medical Genetics and Genomics and the Association for Molecular Pathology. Genet. Med. 17 (5), 405–424. 10.1038/gim.2015.30 25741868 PMC4544753

[B60] Rosewich H. Thiele H. Ohlenbusch A. Maschke U. Altmuller J. Frommolt P. (2012). Heterozygous de-novo mutations in ATP1A3 in patients with alternating hemiplegia of childhood: a whole-exome sequencing gene-identification study. Lancet Neurol. 11 (9), 764–773. 10.1016/S1474-4422(12)70182-5. 22850527

[B61] Ross R. G. Heinlein S. Tregellas H. (2006). High rates of comorbidity are found in childhood-onset schizophrenia. Schizophr. Res. 88 (1–3), 90–95. 10.1016/j.schres.2006.07.006 16916600

[B62] Rudd D. Axelsen M. Epping E. A. Andreasen N. Wassink T. (2015). Childhood-onset schizophrenia case with 2.2 Mb deletion at chromosome 3p12.2-p12.1 and two large chromosomal abnormalities at 16q22.3-q24.3 and Xq23-q28. Clin. Case Rep. 3 (4), 201–207. 10.1002/ccr3.192 25914809 PMC4405302

[B63] Ruzzi L. Gagnoux-Palacios L. Pinola M. Belli S. Meneguzzi G. D’Alessio M. (1997). A homozygous mutation in the integrin alpha6 gene in junctional epidermolysis bullosa with pyloric atresia. J. Clin. Invest. 99 (12), 2826–2831. 10.1172/JCI119474 9185503 PMC508131

[B64] Sagar A. Bishop J. R. Tessman D. C. Guter S. Martin C. L. Cook E. H. (2013). Co-occurrence of autism, childhood psychosis, and intellectual disability associated with a *de novo* 3q29 microdeletion. Am. J. Med. Genet. A 161A (4), 845–849. 10.1002/ajmg.a.35754 23443968 PMC3685481

[B65] Sato S. Cerny R. L. Buescher J. L. Ikezu T. (2006). Tau-tubulin kinase 1 (TTBK1), a neuron-specific tau kinase candidate, is involved in tau phosphorylation and aggregation. J. Neurochem. 98 (5), 1573–1584. 10.1111/j.1471-4159.2006.04059.x 16923168

[B66] Seal J. L. Gornick M. C. Gogtay N. Shaw P. Greenstein D. K. Coffey M. (2006). Segmental uniparental isodisomy on 5q32-qter in a patient with childhood-onset schizophrenia. J. Med. Genet. 43 (11), 887–892. 10.1136/jmg.2006.043380 16763011 PMC2563188

[B67] Sekizawa T. Iwata Y. Nakamura K. Matsumoto H. Suzuki A. Suzuki K. (2004). Childhood-onset schizophrenia and tryptophan hydroxylase gene polymorphism. Am. J. Med. Genet. B Neuropsychiatr. Genet. 128B (1), 24–26. 10.1002/ajmg.b.30009 15211625

[B68] Smedemark-Margulies N. Brownstein C. A. Vargas S. Tembulkar S. K. Towne M. C. Shi J. (2016). A novel *de novo* mutation in ATP1A3 and childhood-onset schizophrenia. Cold Spring Harb. Mol. Case Stud. 2 (5), a001008. 10.1101/mcs.a001008 27626066 PMC5002930

[B69] Soden S. E. Saunders C. J. Willig L. K. Farrow E. G. Smith L. D. Petrikin J. E. (2014). Effectiveness of exome and genome sequencing guided by acuity of illness for diagnosis of neurodevelopmental disorders. Sci. Transl. Med. 6 (265), 265ra168. 10.1126/scitranslmed.3010076 PMC4286868 25473036

[B70] Soueid J. Kourtian S. Makhoul N. J. Makoukji J. Haddad S. Ghanem S. S. (2016). RYR2, PTDSS1 and AREG genes are implicated in a Lebanese population-based study of copy number variation in autism. Sci. Rep. 6, 19088. 10.1038/srep19088 26742492 PMC4705475

[B71] Sporn A. Addington A. Reiss A. L. Dean M. Gogtay N. Potocnik U. (2004). 22q11 deletion syndrome in childhood onset schizophrenia: an update. Mol. Psychiatry 9 (3), 225–226. 10.1038/sj.mp.4001477 14699434

[B72] Sreedharan S. Almen M. S. Carlini V. P. Haitina T. Stephansson O. Sommer W. H. (2011). The G protein coupled receptor Gpr153 shares common evolutionary origin with Gpr162 and is highly expressed in central regions including the thalamus, cerebellum and the arcuate nucleus. FEBS J. 278 (24), 4881–4894. 10.1111/j.1742-4658.2011.08388.x 21981325

[B73] Stephenson J. R. Wang X. Perfitt T. L. Parrish W. P. Shonesy B. C. Marks C. R. (2017). A novel human CAMK2A mutation disrupts dendritic morphology and synaptic transmission, and causes ASD-related behaviors. J. Neurosci. 37 (8), 2216–2233. 10.1523/JNEUROSCI.2068-16.2017. 28130356 PMC5338762

[B74] Szego M. J. Zawati M. H. (2016). Whole genome sequencing as a genetic test for autism spectrum disorder: from bench to bedside and then back again. J. Can. Acad. Child Adolesc. Psychiatry 25 (2), 116–121. 27274747 PMC4879951

[B75] Usiskin S. I. Nicolson R. Krasnewich D. M. Yan W. Lenane M. Wudarsky M. (1999). Velocardiofacial syndrome in childhood-onset schizophrenia. J. Am. Acad. Child Adolesc. Psychiatry 38 (12), 1536–1543. 10.1097/00004583-199912000-00015 10596254

[B76] Vantalon V. Briard-Luginbuhl V. Mouren M. C. (2005). [Fragile X syndrome and very early onset schizophrenia: a female case study]. Arch. Pediatr. 12 (2), 176–179. 10.1016/j.arcped.2004.11.019 15694544

[B77] Veltman J. A. Brunner H. G. (2012). De novo mutations in human genetic disease. Nat. Rev. Genet. 13 (8), 565–575. 10.1038/nrg3241 22805709

[B78] Vourdas A. Pipe R. Corrigall R. Frangou S. (2003). Increased developmental deviance and premorbid dysfunction in early onset schizophrenia. Schizophr. Res. 62 (1–2), 13–22. 10.1016/s0920-9964(02)00429-2 12765738

[B79] Walsh T. McClellan J. M. McCarthy S. E. Addington A. M. Pierce S. B. Cooper G. M. (2008). Rare structural variants disrupt multiple genes in neurodevelopmental pathways in schizophrenia. Sci. 320 (5875), 539–543. 10.1126/science.1155174 18369103

[B80] Wilson N. K. Lee Y. Long R. Hermetz K. Rudd M. K. Miller R. (2011). A novel microduplication in the neurodevelopmental gene SRGAP3 that segregates with psychotic illness in the family of a COS proband. Case Rep. Genet. 2011, 585893. 10.1155/2011/585893 23074677 PMC3447216

[B81] Worth P. F. Giunti P. Gardner-Thorpe C. Dixon P. H. Davis M. B. Wood N. W. (1999). Autosomal dominant cerebellar ataxia type III: linkage in a large British family to a 7.6-cM region on chromosome 15q14-21.3. Am. J. Hum. Genet. 65 (2), 420–426. 10.1086/302495 10417284 PMC1377940

[B82] Yan W. Jacobsen L. K. Krasnewich D. M. Guan X. Y. Lenane M. C. Paul S. P. (1998). Chromosome 22q11.2 interstitial deletions among childhood-onset schizophrenics and “multidimensionally impaired”. Am. J. Med. Genet. 81 (1), 41–43. 9514586

[B83] Yan W. L. Guan X. Y. Green E. D. Nicolson R. Yap T. K. Zhang J. (2000). Childhood-onset schizophrenia/autistic disorder and t(1;7) reciprocal translocation: identification of a BAC contig spanning the translocation breakpoint at 7q21. Am. J. Med. Genet. 96 (6), 749–753. 10.1002/1096-8628(20001204)96:6<749::aid-ajmg10>3.0.co;2-k 11121174

[B84] Yao H. Price T. T. Cantelli G. Ngo B. Warner M. J. Olivere L. (2018). Leukaemia hijacks a neural mechanism to invade the central nervous system. Nat. 560 (7716), 55–60. 10.1038/s41586-018-0342-5 PMC10257142 30022166

[B85] Zhou D. Gochman P. Broadnax D. D. Rapoport J. L. Ahn K. (2016). 15q13.3 duplication in two patients with childhood-onset schizophrenia. Am. J. Med. Genet. B Neuropsychiatr. Genet. 171 (6), 777–783. 10.1002/ajmg.b.32439. 26968334 PMC5069586

